# Micronutrient Status and Dietary Intake of Iron, Vitamin A, Iodine, Folate and Zinc in Women of Reproductive Age and Pregnant Women in Ethiopia, Kenya, Nigeria and South Africa: A Systematic Review of Data from 2005 to 2015

**DOI:** 10.3390/nu9101096

**Published:** 2017-10-05

**Authors:** Rajwinder Harika, Mieke Faber, Folake Samuel, Judith Kimiywe, Afework Mulugeta, Ans Eilander

**Affiliations:** 1 Unilever Research & Development, Vlaardingen, 3130 AC, The Netherlands; Ans.Eilander@unilever.com; 2 Non-communicable Diseases Research Unit, South African Medical Research Council, Cape Town 19070, South Africa; Mieke.Faber@mrc.ac.za; 3 Department of Human Nutrition, University of Ibadan, Ibadan 200284, Nigeria; samuelfolake@yahoo.co.uk; 4 School of Applied Human Sciences, Kenyatta University, Nairobi 43844-00100, Kenya; jokimiywe@gmail.com; 5 Department of Nutrition and Dietetics, Mekelle University, Mekelle 1871, Ethiopia; afework.mulugeta@gmail.com

**Keywords:** iron, anaemia, vitamin A, folate, zinc, iodine, deficiency, intake, women, Africa

## Abstract

A systematic review was conducted to evaluate the status and intake of iron, vitamin A, iodine, folate and zinc in women of reproductive age (WRA) (≥15–49 years) and pregnant women (PW) in Ethiopia, Kenya, Nigeria and South Africa. National and subnational data published between 2005 and 2015 were searched via Medline, Scopus and national public health websites. Per micronutrient, relevant data were pooled into an average prevalence of deficiency, weighted by sample size (WAVG). Inadequate intakes were estimated from mean (SD) intakes. This review included 65 surveys and studies from Ethiopia (21), Kenya (11), Nigeria (21) and South Africa (12). In WRA, WAVG prevalence of anaemia ranged from 18–51%, iron deficiency 9–18%, and iron deficiency anaemia at 10%. In PW, the prevalence was higher, and ranged from 32–62%, 19–61%, and 9–47%, respectively. In WRA, prevalence of vitamin A, iodine, zinc and folate deficiencies ranged from 4–22%, 22–55%, 34% and 46%, while in PW these ranged from 21–48%, 87%, 46–76% and 3–12% respectively. Inadequate intakes of these micronutrients are high and corresponded with the prevalence figures. Our findings indicate that nationally representative data are needed to guide the development of nutrition interventions and public health programs, such as dietary diversification, micronutrient fortification and supplementation.

## 1. Introduction

Micronutrient deficiencies in women of reproductive age (WRA) are known to impair health, pregnancy outcomes and growth as well as the development of their offspring [1,2]. Women are vulnerable to micronutrient deficiencies due to inadequate dietary intake, lack of availability of food, inequitable distribution of food within the same household, lack of knowledge about the importance of dietary diversity and frequent occurrence of infectious diseases [3]. In several developing countries, societal norms and gender-based discrimination require women to put their family members before their own health and nutritional needs, and consequently increase the risk of micronutrient deficiency even further in this population group [3,4]. Thus, WRA in low- and middle-income countries often enter pregnancy malnourished, and the additional demands of pregnancy may further exacerbate micronutrient deficiencies in these pregnant women (PW) [1,2]. 

The most common micronutrient deficiencies in women are iron, vitamin A, iodine, folate and zinc [5]. It is well known that iron deficiency has adverse effects on productivity and cognition in the general population and is the leading cause of anaemia during pregnancy, contributing to 20% of all maternal and perinatal mortality and low birth weight [6,7]. Vitamin A deficiency (VAD) can cause impaired vision (e.g., night blindness) and immune function [8], and may result in preterm birth and infant mortality [9]. Iodine deficiency impairs mental functioning, with an estimated intellectual loss of 7.4 to 15 Intelligence Quotient (IQ) points in infants born to mothers with a poor iodine status [10,11]. Folate deficiency at the time of conception can cause neural tube defects in infants [12]. Zinc deficiency has been suggested as a risk factor with adverse long-term effects on growth, immunity, and metabolic status of surviving offspring [13]. 

Globally anaemia and VAD prevalence in women are decreasing; however, regions of Africa (and Southeast Asia) still have alarming rates of anaemia in WRA, including PW, and the prevalence of VAD is also reported to be high in PW [14,15]. Precise data on iodine, folate and zinc status in African women are lacking; nevertheless, the prevalence of corresponding micronutrient deficiencies based on inadequate intakes is estimated to be high [16]. The high nutritional burden in women has been recognized by the UN Sustainable Development Goals with a target to address the nutritional needs of adolescent girls, as well as pregnant and lactating women, by 2030 [17]. To develop public health strategies and monitor programmes to reach these goals, data on micronutrient status and intake in WRA and PW are essential. For African countries, these data are largely absent and mostly outdated. Therefore, this study aims to perform a systematic review to evaluate recent data on micronutrient status and dietary intake of iron, vitamin A, iodine, folate and zinc in WRA (≥15–49 years) and PW in four of the seven largest and rapidly growing countries in Africa [18,19], including Ethiopia, Kenya, Nigeria and South Africa.

## 2. Methods

### 2.1. Search Strategy

A systematic approach was followed to select all studies with data on iron, vitamin A, iodine, folate and zinc status and intake in Ethiopia, Kenya, Nigeria and South Africa. A literature search was conducted on Medline, Scopus, World Health Organisation (WHO) and The United Nations Children’s Fund (Vitamin and Mineral Nutrition Information System) databases. A combination of the following terms was used to search abstracts and titles: 

(Anaemia OR iron OR vitamin A OR iodine OR folate OR zinc) AND (deficiency OR intake OR status OR prevalence) AND (Ethiopia OR Kenya OR Nigeria OR South Africa) AND (women OR adult OR adolescent OR pregnant). 

Full-text articles were obtained and reviewed to identify those that met the selection criteria below. The reference lists of all articles of interest were checked for additional studies. Websites of public health organizations were searched and local experts were contacted to get access to additional studies and surveys.

### 2.2. Inclusion Criteria

After the initial search, data from different study types including national surveys, population-based observational (cross-sectional or longitudinal) studies, or baseline or control group data from intervention studies were screened to determine eligibility based on the following inclusion criteria:

1. National and subnational surveys or studies reporting data on either anaemia or iron or vitamin A or iodine or folate or zinc status in apparently healthy PW and/or WRA (aged ≥15–49 years) in each country as assessed by the following biomarkers:
  - Iron:
    Anaemia: For WRA Hb < 120 g/L; for PW Hb < 110 g/L. Roughly 50% of anaemia cases are caused by iron deficiency (18), therefore, anaemia prevalence was also included;
    Iron Deficiency (ID): Serum ferritin < 15 μg/L, regardless of correction for inflammation;
    Iron Deficiency Anaemia (IDA): Combination of anaemia and ID [20]
  - VAD: serum retinol < 0.7 nmol/L or (20 μg/dL) [21] for WRA and PW;
  - Folate deficiency: Serum folate < 3 ng/mL (<6.8 nmol/L) severe deficiency and 3–5.9 ng/mL (6.8–13.4 nmol/L) possible deficiency [22];
  - Zinc deficiency: Serum zinc < 10.7 umol/L (70 μg/dL) for WRA and 8.6 umol/L (56 μg/dL) for PW [23];
  - Iodine deficiency: Urinary iodine excretion (UIE) < 100 μg/L for WRA and <150 μg/L for PW [24,25].
2. Dietary intake data of iron, vitamin A, zinc, or folate measured at individual level and for iodine, consumption of iodized salt at household level in each country.
3. Studies and surveys conducted and published 2005–2015.

### 2.3. Data Extraction 

#### 2.3.1. Status Data

For micronutrient deficiencies, we extracted the reported prevalence of anaemia, ID, IDA, VAD, iodine, folate and zinc deficiency when meeting the inclusion criteria. For the biochemical markers of micronutrient status, we extracted the means and when reported, standard deviations (SD) from each data source and separately reported clinical and subclinical deficiencies per micronutrient. For example, the prevalence of clinical signs of VAD (i.e., night blindness, Bitot’s spots, corneal xerosis, xeropthalmia) [21] and iodine deficiency (i.e., goitre) [25] were reported separately from their subclinical signs (e.g., serum retinol and UIE). 

#### 2.3.2. Intake Data

Information on daily dietary intake of iron, vitamin A, folate and zinc in WRA and PW was included as reported. For iodine, little data were available and, therefore, data on household consumption of iodized salt was used. When data was reported as median (and ranges), it was converted to mean by taking an average of median and the interquartile ranges (IQR). For converting the ranges to standard deviation (SD), the difference between high and low IQR was divided by 1.35. When variation was expressed in standard errors of mean (SEM) or confidence intervals, these were converted to SDs.

#### 2.3.3. Data Analysis

When, within a country, more than one study and/or survey was included in the status data of the same micronutrient, the results of these were pooled for each micronutrient and biomarker separately into a weighted mean, which was the weighted average (WAVG) of the sample size of these studies and/or surveys. Therefore, the WAVG is based on all available subnational data and national data. Per country and per micronutrient, the WAVG for PW and WRA were calculated separately. All data were handled and analysed using an Excel spreadsheet (2016).

#### 2.3.4. Calculating Inadequacy of Micronutrient Intakes 

The Estimated Average Requirement (EAR) cut-point method provides a way to estimate the prevalence of inadequate nutrient intake in a population. The proportion of subjects with intake below the EAR was used to estimate the prevalence of inadequate intake of micronutrients in the population. As per Institute of Medicine (IOM) guidelines [26], a conversion factor of 1.6 was used for iron, 1.5 for vitamin A, 1.4 for folate and 1.2 for zinc for calculating EAR from Recommended Daily Allowance (RDA) set by WHO/FAO (Food and Agriculture Organization) [27]. For iron intake in pregnancy, the EAR for pregnancy set by IOM was used [26]. When values for vitamin A intake were reported as beta-carotene, they were converted to retinol activity equivalents (RAE) by dividing with 12 [26]. The bioavailability of 12% was used for dietary iron and the “lowest bioavailability” was used for dietary zinc. For each survey/study, the prevalence of inadequate intake for iron, vitamin A, folate and zinc were estimated by comparing the reported mean and SD to the corresponding EAR, assuming a normal distribution of the data [28]. For iodine, percentage of households consuming inadequately iodized salt (<15 ppm) or non-iodized was included as reported. 

## 3. Results

### 3.1. Data Availability

**Figure 1:**
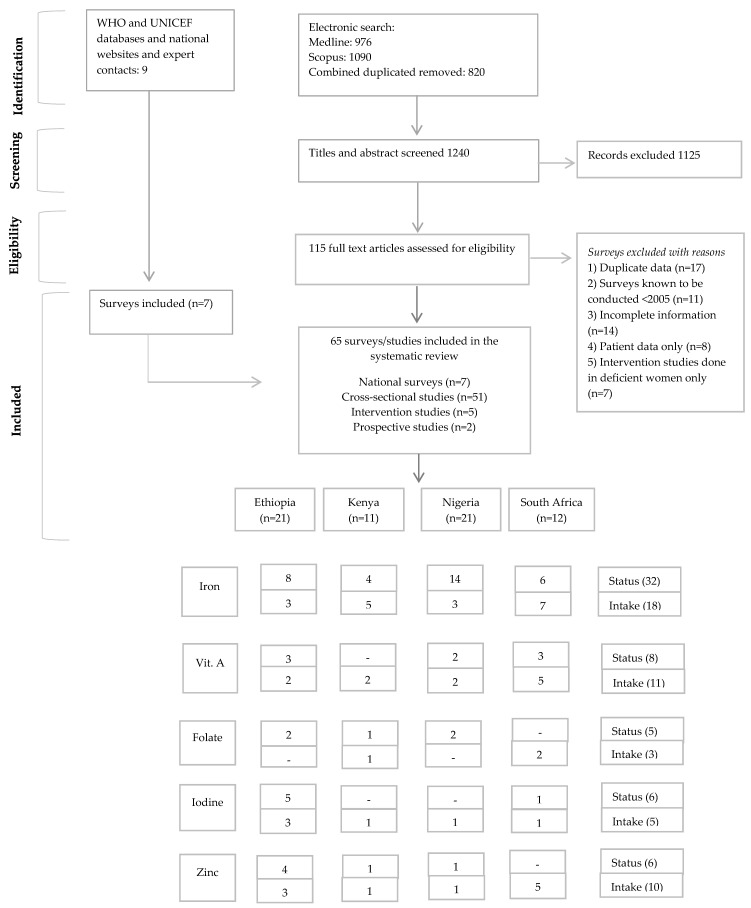
PRISMA flow diagram [36] of the identification of literature for inclusion in this systematic review.

A total of 115 studies and nine surveys were identified from the literature search, whereas only 65 datasets met the inclusion criteria (Figure 1). Out of these, 21 were included for Ethiopia, 11 for Kenya, 21 for Nigeria and 12 for South Africa. The data from Ethiopia are predominantly from rural areas, whereas the data from Kenya, Nigeria and South Africa represent an equal number of rural and urban studies (Table 1). 

Of the 65 data sources, seven were national and 58 were subnational data (51 cross-sectional studies, five intervention studies and two prospective cohorts). For national data, three surveys were from Ethiopia [29,30,31], one from Kenya (iodine data only) [32], and Nigeria (iodine data only) [33] each, and two were from South Africa [34,35]. The majority of the data was on iron status and intake and fewer data was found on vitamin A, iodine, folate and zinc. For 13 studies the sample size was <100; for the remaining studies and surveys it ranged from 100 to 32,079.

### 3.2. Prevalence of Micronutrient Deficiencies in WRA

#### 3.2.1. Status 

**Figure 2:**
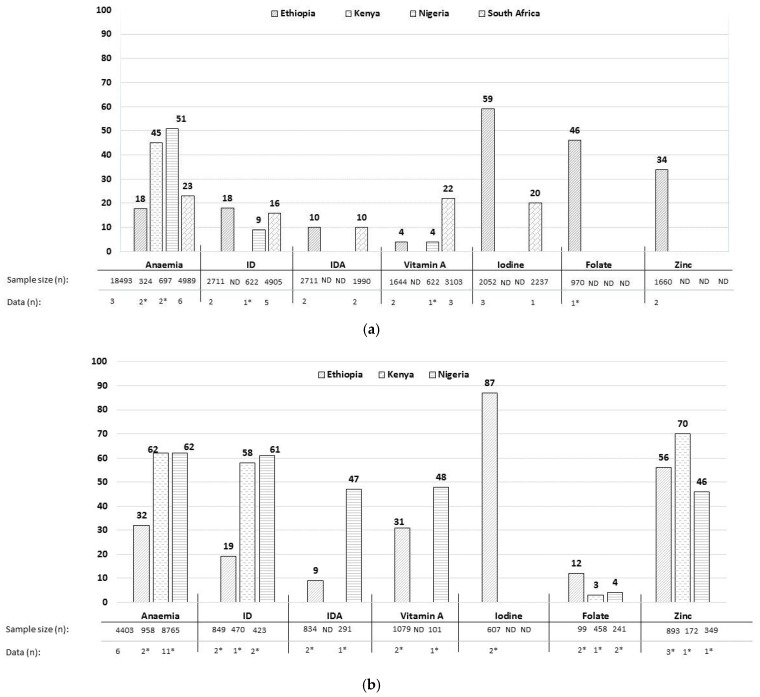
(**a**) Prevalence of micronutrient deficiencies (%) in women of reproductive age in Ethiopia, Kenya, Nigeria and South Africa. The prevalence in percentage (%) are reported as weighted average (WAVG), i.e., an average prevalence that was weighted for the sample size of the studies (subnational data) and national surveys (national data); data (*n*) show number of studies and surveys included; * only subnational data; ID: iron deficiency; IDA: iron deficiency anaemia; ND: no data. (**b**) Prevalence of micronutrient deficiencies (%), in pregnant women in Ethiopia, Kenya and Nigeria. The prevalence in percentage (%) are reported as weighted average (WAVG), i.e., an average prevalence that was weighted for the sample size of the studies (subnational data) and national surveys (national data); data (*n*) show number of studies and surveys included; * only subnational data; ID: iron deficiency; IDA: iron deficiency anaemia; ND: no data.

The most complete data in WRA were found for Ethiopia followed by South Africa, Nigeria and Kenya. Anaemia rates ranged from 18 to 51% (WAVG) in the four countries (Figure 2a). The prevalence of ID ranged from 9% to 18% in Ethiopia, Nigeria and South Africa, and that of IDA was 10% in Ethiopia and South Africa each (Figure 2a). The prevalence of VAD ranged from 4% to 22% (WAVG) in Ethiopia, Nigeria and South Africa (Figure 2a), with no status data for Kenya. Iodine deficiency rates ranged from 59% in Ethiopia to 20% in South Africa (Figure 2a), and no iodine status data were found for Kenya and Nigeria. The prevalence of folate and zinc deficiency was only reported in Ethiopia and was 46% and 34%, respectively (Figure 2a). 

#### 3.2.2. Intake Data

Mean dietary iron intake ranged from 3.8 to 97.8 mg/d (Table 2) and 34–100% of the WRA in Kenya, Nigeria and South Africa had inadequate intakes. Iron intake in Ethiopia was reported to be high (47–97.8 mg/d) and only 8–12% of the WRA had inadequate intake. Mean dietary vitamin A intake ranged from 71 to 2477 μg/d, and 3–100% had inadequate intake (Table 2). Mean dietary folate intake was reported only for South Africa and ranged from 82 to 334 μg/d and 47–98% had inadequate intake (Table 2). Mean dietary zinc intake ranged from 3.8–16.2 mg/d and 23–96% had inadequate intake (Table 2) in Ethiopia, Nigeria and South Africa. The percentage of households not consuming adequately iodized salt (>15 ppm) ranged from 23% to 98% in Ethiopia, Nigeria and South Africa (Table 2). Information on intake of adequately iodized salt was not available for Kenya and reported the intake of non-iodized salt (1% of the households) only. 

### 3.3. Prevalence of Micronutrient Deficiencies in PW

#### 3.3.1. Status

The most complete data in PW were found for Ethiopia, followed by Nigeria and Kenya. No status data for PW from South Africa met the inclusion criteria. The prevalence of anaemia was higher in PW than WRA and ranged from 32% to 62%; 19–61% had ID (Figure 2b). IDA rates were 9% in Ethiopia and 47% in Nigeria (Figure 2b). None of the national surveys reported VAD (based on serum retinol) and the prevalence is therefore based on subnational data only. VAD prevalence was higher in PW than WRA and ranged from 31% to 48% in Ethiopia, and Nigeria (Figure 2b). Iodine deficiency was only reported in Ethiopia, at 87% (Figure 2b). Limited data from five subnational studies were found on folate status, with no national surveys reporting it. Folate deficiency was 12% in Ethiopia, 3% in Kenya and 4% in Nigeria (Figure 2b). Zinc deficiency was at 56% for Ethiopia, 70% for Kenya and 46% for Nigeria (Figure 2b). 

#### 3.3.2. Intake Data

Mean dietary iron intakes ranged from 9.6 to 28.3 mg/d and up to 99% of the PW had inadequate intakes (Table 2). Mean vitamin A intakes ranged from 436 to 2645 μg/d and 3–48% had inadequate intakes (Table 2) in Kenya, Nigeria and South Africa. Mean dietary folate intake was reported only in Kenya at 364 μg/d (Table 2). Mean dietary zinc intake ranged from 5.4 to 9.4 mg/d and 84 to 99% had inadequate intakes in Ethiopia, Kenya and South Africa (Table 2). More than 90% of households with PW consumed inadequately iodized salt in Ethiopia; no specific data on iodized salt consumption in households with PW were found in the other three countries (Table 2).

### 3.4. Biomarker Data in WRA and PW

Mean haemoglobin concentration ranged from 92 to 147 g/L and mean serum ferritin concentration from 9 to 85 μg/L (Supplementary Figure S1a,b) in PW and WRA. The prevalence of night blindness was reported in PW at 1.1–1.5% (Supplementary Table S1) and serum retinol ranged from 0.84 to 1.49 μmol/L (Supplementary Figure S2a,b) in WRA and PW. UIE ranged from 17 to 188 μg/L (Supplementary Figure S3) and goitre prevalence was reported in Ethiopia only and ranged from 16% to 85% in PW and WRA (Supplementary Table S2). Mean serum folate ranged from 12.6 to 31.3 ng/mL (Supplementary Figure S4) and mean serum zinc concentration ranged from 6.6 to 12.5 μmol/L in PW and WRA (Supplementary Figure S5).

## 4. Discussion

### 4.1. Main Findings and Their Significance

This systematic review, based on national and subnational data published from 2005 to 2015, provides an overview of the prevalence of micronutrient deficiencies in WRA and PW in the four African countries. Anaemia rates ranged from 18% to 51% in WRA in the four countries, ID ranged from 9% to 16% and IDA was at 10%. The prevalence of VAD deficiency in WRA was highest in South Africa at 22%, while that of iodine deficiency was highest in Ethiopia (59%) (Figure 2a). Data on folate (46%) and zinc (34%) deficiencies were only available for Ethiopian WRA (Figure 2a). As expected, the prevalence of anaemia in PW was high, ranging from 32% to 62%, while that of ID and IDA ranged from 19% to 61% and 9–47%, respectively (Figure 2b). Based on limited data, the prevalence of folate deficiency was low in PW (<12%), while zinc deficiency and VAD were high (>30%) (Figure 2b). Iodine deficiency in PW was reported at 87% in Ethiopia only. Notably, no micronutrient status data in PW from South Africa were found. Data on inadequate intake of iron, vitamin A, folate, zinc and household consumption of iodized salt largely corresponded with the prevalence figures for micronutrient deficiencies. 

### 4.2. Strengths and Limitations of This Study

This systematic review is the first to provide an overview of both status and dietary intakes of iron, vitamin A, iodine, folate and zinc in WRA and PW in Ethiopia, Kenya, Nigeria and South Africa. A strength of this review is the use of national and subnational data, which were pooled to determine the prevalence of deficiency in WRA and PW. The restriction of the studies to the years 2005–2015 increases the possibility of data being reflective of the current situation, thereby increasing the validity of this study. This review may help guide public health practitioners and policy makers in advocating for public health strategies to prevent micronutrient deficiencies in WRA and PW, especially where nationally representative data are lacking.

However, there are a few limitations to this systematic review. Most limitations are mentioned in a similar publication on children (0–19 years) from the four countries [95] and will not be discussed again. Some limitations specifically related to this review are noted here. Firstly, prevalence estimates from subnational data can either over- or underestimate the national prevalence of micronutrient deficiencies, as large regional differences in micronutrient status may exist within countries. This is especially the case for data on PW and for countries such as Kenya and Nigeria, where subnational data provided substantial weight to the overall figures on micronutrient deficiencies due to a lack of national data. Nevertheless, this bias has been minimised by removing the data that included deficient populations only (Figure 1). Secondly, for folate, iodine, zinc and vitamin A (PW) very limited data were found, and therefore the WAVG for these micronutrients may not represent the national situation. Lastly, intakes from supplements, such as for iron and folate from common supplementation programs in PW, were not considered by most intake data studies. Therefore, figures for inadequate intakes are crude approximations and should be interpreted as such. 

### 4.3. Findings on Micronutrient Status and Intake in WRA

The prevalence of anaemia, 18–51% in WRA, is a moderate to severe public health problem as per the WHO criteria [20]. Stevens et al. estimated (using data from 1995–2011) that Central and West Africa had the highest anaemia prevalence at about 50% in WRA [15], which is in line with our findings. However, a recent systematic review from 21 African countries (Ethiopia is the only country in common with our review) reported an overall anaemia prevalence of 23% (range 12.5–36%) based on 2003–2010 DHS data in WRA (including PW) [96]. The overall lower anaemia prevalence in this review could be due to the lower anaemia rates in these countries or the type of data (national vs. subnational data from high endemic regions) included. About half of all anaemia is estimated to be attributable to iron deficiency, depending on the geographic and disease environment. Much of the other half is caused by infectious diseases and deficiencies of other micronutrients [20]. Our data on low iron intakes correspond with the figures for anaemia and iron deficiency. However, for Ethiopia iron intake was relatively high at 47.2–97.8 mg/d [29,38], whereas the prevalence of ID in Ethiopia was comparable to other countries (Figure 2a). This high intake is most likely attributed to contaminant iron and consumption of teff [97], which is rich in iron but has poor bioavailability. 

The prevalence of VAD is particularly high in South Africa at 22% vs. 4% in Ethiopia and Nigeria. Similar findings of higher VAD prevalence are also reported in 0–19-year-old South African children compared to the other three countries [95], implying that VAD is a problem of public health significance in South Africa. Vitamin A intakes had large variation, from very low intakes of 71 ug/d in Ethiopia to high intakes of 2477 ug/d in Nigeria; these intakes could be influenced by seasonal variation, mandatory vitamin A fortification policies or differences in intake of vitamin-A-rich organ meat [98,99]. 

Data on the prevalence of iodine deficiency in WRA were only found for Ethiopia (59%) and South Africa (20%) and were partly consistent with households not consuming adequately iodized salt (23–98%) in these countries. Although household use of adequately iodized salt is used as proxy for iodine intake, certain processed foods containing iodized salt could significantly contribute to iodine intake [100]; this may explain some of the discrepancies between iodine status in WRA and household use of adequate iodized salt. 

A decrease in folate deficiencies after the introduction of folic acid fortification of staple food has been reported in population surveys by many countries, including South Africa [101]. However, our review indicates that there are limited data available to confirm these reductions. Ethiopia is yet to implement mandatory fortification of staples with micronutrients including folic acid, which could explain the high folate deficiency rate of 46% in WRA in Ethiopia.

Data on the prevalence of zinc deficiency were found for Ethiopia only (34%). Estimates based on the bioavailability of zinc, physiological requirements and predicted zinc absorption suggest that inadequate zinc intakes occur in over 25% of the population in Southeast Asia and Africa [102]. Thus, the prevalence of zinc deficiency could be substantial (>20%) in the included African countries with no data currently available. 

### 4.4. Findings on Micronutrients Status and Intake in PW

For PW, we did not find any status data from South Africa that met the inclusion criteria (as most data were from before 2005), thus the findings are largely applicable to the other three countries. The prevalence of anaemia in PW of 32–61% is a moderate to severe public health problem as per the WHO criteria [20]. These findings are in line with a recent review that estimated anaemia prevalence in PW > 50% in Central and West Africa [15]. Africa also has the highest prevalence of IDA in pregnant women [15]; this resonates with the findings on IDA at 47% in Nigeria. However, it was only 9% in Ethiopia. It should be noted that the prevalence of ID and IDA in the current review may be underestimated because of uncertainty about the correction of ferritin levels for inflammation and/or infection [103]. Because of increased needs during pregnancy, the recommendation for iron intake increases by 50% compared to that in WRA, and therefore pregnant women are more vulnerable to inadequate iron intake, which is also indicated in our study. However, it is unclear from several intake studies whether iron intake from supplements (as are often prescribed for PW) was considered. 

Next to young children, PW are at the highest risk for VAD due to their increased demands for vitamin A and the potential health consequences associated with its deficiency during this life stage. The data on VAD rates (31–48%) based on serum retinol in PW in this review are based on three subnational studies and are higher than those reported by the WHO for the African region (14% (9.7–19%; 95%CI)) [104]. However, when comparing clinical data on night blindness as an indicator for VAD (see Table S1), the data show a lower prevalence (1.1 to 1.5%) compared to WHO reports for Africa (9.8%) [104]. 

Except for Ethiopia, we found no iodine status data in PW. This is of concern as PW are particularly vulnerable to iodine deficiency due to their increased needs. Assessments of median UIE in 6–12-year-old children could be used to estimate the iodine status of the general adult population; these, however, cannot be extrapolated to PW [24]. Therefore, assessments of iodine status in PW are highly recommended. 

The prevalence of folate deficiency in Kenya, Nigeria and Ethiopia was at 3%, 4% and 12%, respectively. Nevertheless, the included studies are small and not nationally representative, with the exception of one study from Ethiopia [48]. Dietary data on folate intake is limited; however, the intake of folic acid supplements during pregnancy could explain the lower level of folate deficiency in these countries . Nevertheless, our data on prevalence of folate deficiency are in contrast to the high prevalence of neural tube defects in sub-Saharan Africa, with figures ranging from 0.16 to 7 per 1000 live births [105]. Therefore, more data are needed to conclude whether folate status is sufficient in both PW and WRA in African countries. 

Similar to folate, the data on zinc are limited and mostly based on subnational data, with deficiency reported at 46% to 70% in PW in Ethiopia, Kenya and Nigeria. Zinc deficiency is common in developing countries and low maternal circulating zinc concentrations have been associated with pregnancy complications [13]. As diet is the main factor that determines zinc status [106], inadequate intake of zinc due to a primarily plant-based diet is by far the most likely cause of zinc deficiency in these populations. It is widely acknowledged that many PW do not meet the recommended intakes of zinc [107] and our data also show that 84% to 97% of PW have inadequate intake.

### 4.5. Implications for Policies and Programmes

It is critical that WRA enter pregnancy with the best possible macro- and micronutrient status and receive adequate nutrition during pregnancy for their health and for the wellbeing of their offspring. The adverse consequences of a poor maternal diet are even more severe in adolescent pregnancy; however, data on adolescents are largely lacking. To facilitate the nutritional health and wellbeing of PW and WRA in developing countries, population-level sustainable and globally standardized dietary monitoring is essential. In the absence of national dietary surveys, conducting regular smaller surveys in different regions in the country can also provide important information and insights to policy makers and governments. 

As the majority of the population in these countries relies on staple foods, the fortification of staples (e.g., flour, oils, salt) has been implemented by various African countries; nevertheless, progress is not consistent across countries. South Africa and Nigeria have been practising mandatory fortification of flours for more than a decade, Kenya has recently started such an initiative, and Ethiopia is in the planning phase. Moreover, it is important to note that with legislation on mandatory food fortification, the quality of fortified foods is not automatically guaranteed as compliance to legislation is found to be low [108]. In addition to food-based programmes, daily or intermittent iron supplementation, alone or together with other micronutrients, should be strengthened, especially for PW to improve their intake. Currently, coverage and compliance to supplementation programmes throughout pregnancy is far from optimum [32] despite clear recommendations by the WHO and national bodies [109,110]. Moreover, the coverage of supplementation programs has been worse in women of poor socioeconomic status and with unplanned pregnancies (especially in adolescents). 

Therefore, the fortification of other commonly consumed foods that are widely available and affordable, such as condiments and cooking aids (including seasonings and bouillon cubes) has the potential to improve the nutritional status of women and their families and has the advantage of reaching women prior to pregnancy [111,112]. Recent studies have shown that bouillon cubes fortified with vitamin A or iodine can make a large contribution to the recommended daily intake of these micronutrients in poor population groups and can help decrease the prevalence of micronutrient deficiencies [100,113]. Next to this, dietary diversification and improved access to foods that have high micronutrient bioavailability, including animal products, are also important strategies. 

## 5. Conclusions

In conclusion, the available data indicate that the prevalence of anaemia and iron deficiencies in PW and WRA in Ethiopia, Kenya, Nigeria and South Africa are a public health concern based on the WHO criteria. Limited data indicate that vitamin A, iodine, zinc and folate nutrition may also be suboptimal in both WRA and PW in these countries. Underlying these deficiencies are inadequate dietary intakes, as indicated by the available studies. Therefore, nationally representative data on micronutrient status and intake of especially vitamin A, iodine, zinc and folate in PW and WRA are urgently needed to further guide the development of public health programmes and monitor the impact of these programmes on a regular basis. These programs should focus on improving micronutrient intake through stimulating dietary diversity and the fortification of commonly consumed and affordable food products. In addition, supplementation programmes need to be scaled up for pregnant women. 

## Figures and Tables

**Table 1:** Characteristics of the studies and surveys included from Ethiopia, Kenya, Nigeria and South Africa.

	Reference	Year of Survey	Study Location	State/Province	Age	*n*	Study Design	Data Included
Ethiopia								
**National data**								
	Ethiopia Public Health Institute, 2013 [29]	2011	Nationwide	National	15–49 years	7908	National Food consumption survey	Iron, vitamin A, zinc intake
	Ethiopian Public Health Institute, 2015 [30]	2015	Nationwide	National	-	1741	National (micronutrient) survey	Iron, vitamin A, zinc status
	DHS, 2011 [31]	2011	Nationwide	National	15–49 years	15782 (WRA) + 1173 (PW)	Demographic and Health Survey (DHS)	Iron status
**Subnational data**								
	Abuye, 2008 [37]	2008 *	5 states, cluster sampling method was applied to select the study population	Amhara, Tigray, Oromiya, SNNP & Benishangul-Gumuz	-	6960	Cross-sectional study	Iodine status
	Amare 2012 [38]	2005	Gondar city (urban setting; two-stage probability sampling method was used for selecting the study population)	Amhara	>18 years	356	Cross-sectional study	Iron and vitamin A intake
	Abriha, 2014 [39]	2014	Mekelle town (urban setting)	Amhara	16–40 years pregnant (all trimesters)	619	Cross-sectional study	Iron status
	Bogale, 2009 [40]	2007	Sidama zone (rural area)	Sidama	28 years	99	Cross-sectional study	Iodine status and household consumption of iodized salt
	Ersino, 2013 [41]	2009	Tulu Health Center (rural area)	Sidama	27.7 ± 5.6 years pregnant †	172	Cross-sectional study	Iodine status and household consumption of iodized salt
	Gebremedhin, 2011 [42]	2011	Sidama zone (rural area with primarily subsistent farming)	Sidama	15–≥35 years pregnant (all trimesters)	700	Cross-sectional study	Zinc status
	Gebremedhin, 2014 [43]	2011	Sidama (rural area with primarily subsistent farming)	Sidama	15–49 years pregnant (all trimesters)	750	Cross-sectional study	Iron status
	Gebreselassie, 2013 [44]	2011	Kebeles of Sidama zone (rural area)	Sidama	~29 years pregnant (all trimesters)	700	Cross-sectional study	Vitamin A status
	Gebreegziabher, 2013 [45]	2009	Rural communities of Sidama zone,	Sidama	30.8 ± 7.8 years	202	Cross-sectional study	Iodine status
	Gibson, 2008 [46]	2008 *	Sidama (rural area with primarily subsistent farming)	Sidama	28 years pregnant †	99	Cross-sectional study	Iron, vitamin A, folate status iron and zinc intake
	Haidar, 2009 [47]	2005	Tigray; Affar; Amhara; Oromiya; Benishangul-Gumuz; Southern Nations, SNNP; Harari regions; Addis Ababa and Dire Dawa city administrations (80% of the population was from rural setting)	9 regions	15–49 years	970	Cross-sectional study	Iron status
	Haider, 2010 [48]	2005	Tigray; Affar; Amhara; Oromiya; Benishangul-Gumuz; Southern Nations, SNNP; Harari regions; Addis Ababa and Dire Dawa city administrations (80% of the population was from rural setting)	9 regions	15–49 years	970	Cross-sectional study	Folate status
	Hambidge, 2006 [49]	2006 *	Alamura (rural area with resource poor setting)	Rift Valley	27.8 ± 4.7 years pregnant (3rd trimester)	17	Cross-sectional study	Zinc intake
	Joray, 2015 [50]	2010	Sidama (rural area)	Sidama	18–50 years	35	Baseline data of an intervention trial	Zinc status
	Kedir, 2013 [51]	2010	Haramaya district (primarily rural area)	Oromia	25 ± 5 years	1678	Cross-sectional study	Iron status
	Kedir, 2014 [52]	2012	Haramaya district (10 administrative rural unit rural)	Oromia	27 ± 5.9 years pregnant (all trimesters)	435	Cross-sectional study	Iodine status
	Mulu, 2011 [53]	2005	University of Gondar Hospital	Northwest	16–45 years	25	Cross-sectional study	Vitamin A status
	Stoecker, 2009 [54]	2009 *	Sidama region (rural area with primarily subsistent farming)	Sidama	27.7 ± 4.7 years pregnant (2nd & 3rd trimester)	99	Cross-sectional study	Iron status
Kenya								
**National data**								
	DHS, 2015 [32]	2014	Nationwide	National	15–49 years	34139 (HH)	Demographic and Health Survey (DHS)	Household consumption of iodized salt
**Subnational data**								
	Adongo, 2013 [55]	2013 *	Kalacha Location, Marsabit County (pastoral area with livestock)	Eastern	15–49 years	224	Cross-sectional survey	Iron status and intake
	Gitau, 2008 [56]	2008 *	Makongeni, Thika District	Central	-	100	Cross-sectional study	Iron status
	Kamau-Mbuthia, 2007 [57]	2004–2005 **	Provincial General Hospital, Nakuru, (urban area)	Rift valley	~25 years pregnant †	716	Cross-sectional study	Iron and zinc intake
	Mwangi, 2014 [58]	2011–2012	Nyanza Province (rural area)	Nyanza	15–45 years pregnant (2nd trimester)	470	Baseline of intervention trial	Iron status
	Mitheko, 2015 [59]	2015 *	Naivasha	Nakuru	pregnant †	172	Cross-sectional study	Zinc status
	Ouma, 2006 [60]	2003–2005 **	3 hospitals in Nyanza province (primarily rural area)	Nyanza	<20 years pregnant (2nd & 3rd trimester)	488	Baseline of intervention trial	Iron status
	Othoo, 2014 [61]	2014 *	Ndhiwa Maternal and Child Health clinic	Nyanza	pregnant †	162	Cross-sectional study	Iron and vitamin A intake
	Shipala, 2012 [62]	2008	2 health facilities in Bungoma	Western	<19 years pregnant †	384	Cross-sectional study	Iron and folate intake
	Van Eijk, 2008 [63]	2003–2005 **	3 hospital in Nyanza province	Nyanza	<20 years pregnant †	458	Baseline of intervention trial	Folate status
	Waswa, 2011 [64]	2011 *	Moi University, Eldoret Town (urban area)	Nyanza	20–25 years	260	Cross-sectional survey	Iron and vitamin A status
Nigeria								
**National data**								
	DHS, 2008 [33]	2008	Nationwide	National	15–49 years	32079 (HH)	Demographic and Health Survey	Household consumption of iodized salt
**Subnational data**								
	De Moura, 2015 [65]	2011	31 local government area in Akwa-Ibom (primarily rural area)	Akwa-Ibom	18–49 years	622	Cross-sectional survey	Iron, vitamin A and zinc intake
	Dim, 2007 [66]	2005	University of Nigeria teaching hospital, Enugu city (urban)	Enugu	21 ± 7 years pregnant (all trimesters)	530	Cross-sectional study	Iron status
	Dim, 2014 [67]	2012	University of Nigeria teaching hospital, Enugu city (urban)	Enugu	16–45 years pregnant (all trimesters)	200	Cross-sectional study	Iron status
	Ezugwu, 2013 [68]	2009–2010	University of science and technology teaching hospital, Enugu city (urban)	Enugu	25 ± 5 years pregnant (all trimesters)	1306	Cross-sectional study	Iron status
	Idowu, 2005 [69]	2005 *	Ogun	Abeokuta	>15 years pregnant (all trimesters)	477	Cross-sectional study	Iron status
	Kagu, 2007 [70]	2005–2006	Federal Medical Center, Nguru (rural area with subsistence farming and fishing)	Yobe	13–48 years pregnant (all trimesters)	1040	Prospective study	Iron status
	Miri-Dashe, 2014 [71]	2014 *	Plateau State Specialist Hospital, Jos (urban area)	Plateau	18–65 years pregnant (all trimesters)	125 (WRA) + 134 (PW)	Cross-sectional study	Iron status
	Nwizu, 2011 [72]	2011 *	Aminu Kano Teaching hospital, Kano (urban area)	Kano	>15 years pregnant (all trimesters)	300	Cross-sectional study	Iron status
	Okwu & Ukoha 2008 [73]	2008*	Owerri (majority urban and some rural areas were included)	Imo	>18 years pregnant †	1387	Cross-sectional study	Iron status
	Olatunbosun, 2014 [74]	2012	University of Uyo Teaching Hospital, Uyo City (urban)	Akwa Ibom	17–45 years pregnant (2nd & 3rd trimester)	400	Cross-sectional study	Iron status
	Otemuyiwa, 2012 [75]	2012 *	Adekunle Ajasin University, Akungba-Akoko & Obafemi Awolowo University (rural), Ile-Ife (urban)	Osun	15–35 years	203	Cross-sectional study	Iron intake
	Obasi, 2013 [76]	2013 *	Federal teaching hospital Abakaliki & st Vincent Hospital Ndubia (rural)	Ebonyi	15–40 years pregnant (2nd trimester)	295	Cross-sectional study	Iron status
	Ofojekwu, 2013 [77]	2013 *	Federal School of Medical Laboratory Science and the School of Nursing and Midwifery in Jos City (urban)	Plateau	19–30 years	46	Cross-sectional study	Iron status
	Olubukola, 2011 [78]	2008	University college hospital & Adeoyo maternity hospital in Ibadan (urban)	Oyo	27 years pregnant (all trimesters)	2702	Cross-sectional Study	Iron status
	Shu & Ogbodo 2005 [79]	2005 *	University of Nigeria teaching hospital, Enugu City (urban)	Enugu	18–42 years pregnant (all trimesters)	74	Cross-sectional study	Iron status
	Ugwuja, 2009 [80]	2007–2008	Federal medical Centre, Abakaliki (rural)	Ebonyi	15–40 years pregnant (2nd trimester)	349	Cross-sectional study	Iron status
	Ugwuja, 2010 [81]	2007–2008	Fedearl medical centre, Abakaliki (rural, with mixed population)	Ebonyi	15–40 years pregnant (2nd trimester)	349	Cross-sectional study	Zinc status
	VanderJagt, 2011 [82]	2011 *	Jos teaching university hospital, Jos City (urban)	Plateau	28 ± 6.1 years pregnant (all trimesters)	143	Cross-sectional study	Folate status
	VanderJagt, 2009 [83]	2009 *	Jos teaching university hospital, Jos city (urban)	Plateau	27.4 ± 5.4 years pregnant (3rd trimester)	98	Cross-sectional study	Folate status
	Williams, 2008 [84]	2006	University of Calabar Teaching Hospital, Calabar (urban)	Cross River State	pregnant (all trimesters)	101	Cross-sectional study	Vitamin A intake and status
South Africa								
**National data**								
	NFCS, 2007 [34]	2005	Nationwide	National	16–35 years	~2400	National survey	Iron, vitamin A and iodine status and household consumption of iodized salts
	Shisana, 2014 [35]	2012	Nationwide	National	16–35 years	~1300	National health and nutrition survey	Iron and vitamin A status
**Subnational data**								
	Dolman, 2013 [85]	2005/12	North West (rural and urban)	North West	~47 years	1068	Prospective Urban and Rural Epidemiological (PURE) study	Iron, vitamin A, folate and zinc intake
	Faber, 2005 [86]	2005*	Ndunakazi (rural)	KwaZulu-Natal	29.7 ± 7.6 years	118	Cross-sectional survey	Iron and vitamin A status
	Gichohi-Wainaina, 2015 [87]	2005	North West (rural and urban)	North West	32–86 years	678	PURE cohort study (baseline data)	Iron status
	Hattingh, 2008 [88]	2008 *	Mangaung (urban)	Bloemfontein	25–34 years	279	Cross-sectional survey	Iron, vitamin A and zinc intake
	Kolahdooz, 2013 [89]	2013 *	Empangeni (rural)	KwaZulu-Natal	19–50 years	40	Cross-sectional study	Iron, vitamin A and zinc intake
	Lawrie, 2008 [90]	2008 *	Workers at Helen Joseph and Coronation hospital, Gauteng (urban)	Gauteng	>18 years	631	Cross-sectional study	Iron status
	Mostert, 2005 [91]	2005 *	Dikgale primary health care clinic, Limpopo (rural, resource poor setting)	Limpopo	13–40 years pregnant †	46	Cross-sectional study	Iron, vitamin A and zinc intake
	Oldewage-Theron, 2011 [92]	2011 *	Informal settlement in Vaal region (a peri-urban area, resource poor setting)	Gauteng	-	426	Cross-sectional study	Iron, vitamin A, folate and zinc intake
	Oldewage-Theron, 2014 [93]	2008–2009	Qwa-Qwa (rural households)	Free State	39.8 ± 13.5 years	83	Single system case study (baseline data)	Iron Status and intake
	Pisa, 2012 [94]	2005	North West (rural and urban)	North West	>35 years	1264	Prospective Urban and Rural (PURE) Epidemiological study (baseline data)	Iron intake

* Year of survey not available, hence year of publication was assumed to be the year of survey; CG-control group; HH-households; WRA-women of reproductive age; PW-pregnant women; ** data collection was done in 2002–2005, but were included due to scarce data in Kenya; † trimester not mentioned in the study; for pregnant women, unless data was reported for only one trimester, an average prevalence/intake of the two or three trimesters was included.

**Table 2:** Intake of micronutrients and iodized salt intake in women of reproductive age and pregnant women in Ethiopia, Kenya, Nigeria and South Africa.

Country	Reference	Age Group	*n*	Dietary Information	Iron		Vitamin A		Folate		Zinc		Iodine
					mg/d	Inadequate Intake (%)	μg RE/d	Inadequate Intake (%)	μg/d	Inadequate Intake (%)	mg/d	Inadequate Intake (%)	Households not Consuming Adequately Iodized Salt (%)
Women of reproductive age													
Ethiopia	Amare, 2012 [38]	>18 years	255	24 h dietary recall	97.8 (54.6)	8%	216(25)	100%					
	Bogale, 2009 [40]	28 years	99	Plasma mass spectrometer for iodine content of salt									98%
	Ethiopian Public Health Institute, 2015 [30]	households	3221	Titration method									74%
	NFC Survey, 2013 ^ [29]	15–49 years	7908	24 h dietary recall	47.2 (33.8)	12%	71 (129) †	99%			7.6 (5.7)	53%	
Kenya	Adongo, 2013 [55]	15–49 years	224	24 h dietary recall	11.8 (5.2)	94%							
	DHS, 2014 [32]	households	34139	Rapid test kit for iodine content of salt									1% *
	Waswa, 2011 [64]	20–25 years	260	FFQ	12.6 (6.6)	86%	368 (287)	53%					
Nigeria	De Moura, 2015 ^ [65]	>18 years	579	24 h dietary recall	11.2 (4)	98%	2477 (961)	3%			11.2 (4)	23%	
	DHS, 2008 [33]	households	32079	-									48%
	Otemuyiwa, 2013 [75]	15–35 years	203	FFQ	13.5 (4.5)	92%							
South Africa	Dolman, 2013	47 years	1068	FFQ	11.5 (5.2)	94%	657 (495)	28%	334(162)	47%	8.6 (3.8)	46%	
	Kolahdooz, 2013 [89]	19–50 years	40	24 h dietary recall	24 (10)	45%	216 (336) †	66%			8.3 (3.6)	49%	
	Hattingh, 2008 ^ [88]	25–34 years	279	FFQ	26.7 (15.9)	34%	2221(1472)	11%			16.2(9.3)	20%	
	NFCS, 2007 [34]	16–35 years	2237	Iodometric titration method for iodine content of salt									23%
	Pisa, 2012 [94]	>35 years	1264	QFFQ	12.5 (9.1)	79%							
	Oldewage-Theron, 2014 [93]	19–75 years	84	24 h recall	6.4								
	Oldweage-Theron, 2011 [92]	na	426	24 h recall	3.8 (2)	100%	176 (617)	61%	82 (103)	98%	3.8 (2.4)	96%	
Pregnant women													
Ethiopia	Ersino, 2013 [41]	27.7 ± 5.6 years	172	Plasma mass spectrometer for iodine content of salt									>90%
	Gibson, 2008 [46]	28 years	99	1 day weigh record	28.3						5.4		
	Hambidge, 2006 [49]	27.8 ± 4.7 years	17	24 h weigh record							6 (3.2)	97%	
Kenya	Kamau-Mbuthia, 2007 [57]	~26 years	716	24 h dietary recall	16.1						9.4		
	Shipala, 2012 [62]	<19 years	384	FFQ	26.8				364				
	Othoo, 2014 [61]	21–25 years	162	Semi-structured Questionnaire	18.5		436						
Nigera	Williams, 2008 [84]	-	101	24 h dietary recall			2645 (189)	3%					
South Africa	Mostert, 2005 [91]	13–40 years	46	24 h dietary recall	9.6 (4.3)	99%	574 (428)	48%			8.1 (4.3)	84%	

Intake data presented as mean (SD); ^ Data were reported as median and IQR, but converted to mean; * households consuming iodized salts; † vitamin A reported RAE (retinol activity equivalent).FFQ-Food Frequency Questionnaire; QFFQ- Quantitative Food Frequency Questionnaire.

## Supplementary Materials

The following are available online at http://www.mdpi.com/2072-6643/9/10/1096/s1, Figure S1: Mean (SD) haemoglobin (g/L) and serum ferritin (µg/L) concentration in women of reproductive years (a) and pregnant women (b) in four African countries., Figure S2: Mean (SD) Serum retinol (µmol/L) concentration in women of reproductive age group (a) and pregnant women (b) in four African countries., Figure S3: Median Urinary Iodine Excretion (µg/L) in women of reproductive age and pregnant women in Ethiopia and South Africa., Figure S4: Mean (SD) Serum folate (ng/L) concentration in women of reproductive age (WRA) and pregnant women in Ethiopia and Nigeria, Figure S5: Mean (SD) Serum zinc (µmol/L) concentration in women of reproductive age (WRA) and pregnant women in Ethiopia, Kenya and Nigeria., Table S1: Clinical signs of VAD, night blindness in pregnant women., Table S2: Goiter prevalence in women of reproductive age and pregnant women in Ethiopia.
