# Supplementary material for: Micronutrient Status and Dietary Intake of Iron, Vitamin A, Iodine, Folate and Zinc in Women of Reproductive Age and Pregnant Women in Ethiopia, Kenya, Nigeria and South Africa: A Systematic Review of Data from 2005 to 2015
Source: Nutrients. 2017 Oct 5;9(10):1096. doi: 10.3390/nu9101096 (PMC5691713; doi:10.3390/nu9101096)
Supplement: Supplementary file 1 [file nutrients-09-01096-s001.docx]

South Africa

Nigeria

Kenya

Ethiopia

**Figure S1a.** Mean (SD) haemoglobin (g/L) and serum ferritin (µg/L) concentration in women of reproductive years in four African countries.

Kenya

Nigeria

Ethiopia

**Figure S1b.** Mean (SD) haemoglobin (g/L) and serum ferritin (µg/L) concentration in pregnant women in Ethiopia, Kenya and Nigeria.

**Figure S2a.** Mean (SD) serum retinol (µmol/L) concentration in women of reproductive age group in four African countries.

**Figure S2b.** Mean (SD) Serum retinol (µmol/L) concentration in pregnant women in four African countries.

**Figure S3.** Median urinary iodine excretion (µg/L) in women of reproductive age and pregnant women in Ethiopia and South Africa.

WRA

**Figure S4.** Mean (SD) serum folate (ng/L) concentration in women of reproductive age (WRA) and pregnant women in Ethiopia and Nigeria.

WRA

**Figure S5.** Mean (SD) serum zinc (µmol/L) concentration in women of reproductive age (WRA) and pregnant women in Ethiopia, Kenya and Nigeria.

**Table S1.** Clinical signs of VAD, night blindness in pregnant women.

| **Countries** | **Survey** | **Night blindness** |
| --- | --- | --- |
| Nigeria [33] | DHS 2008 | 1.1% |
| Kenya [32] | DHS 2008-09 | 1.5% |

**Table S2.** Goiter prevalence in women of reproductive age and pregnant women in Ethiopia.

| **Country** | **Reference** | **population** | **Goiter** |
| --- | --- | --- | --- |
| Ethiopia | Abuye, 2008 [37] | women | 39% |
|  | Bogale, 2009 [40] | women | 85% |
|  | Gebreegziabher, 2013 [45] | women | 16% |
|  | Ersino, 2013 [41] | pregnant women | 49% |
